# Prognostic Impact of Ectopic Fat Deposition within Psoas Muscle in Stage IV Gastric Cancer Patients Undergoing Systemic Chemotherapy

**DOI:** 10.7150/jca.78407

**Published:** 2022-10-17

**Authors:** Takako Ikegami, Hiroki Nishikawa, Masahiro Goto, Fukutaro Shimamoto, Tetsuji Terazawa, Toshifumi Yamaguchi, Eiki Yamasaki, Ken Asaishi, Shiro Nakamura, Kazuhide Higuchi

**Affiliations:** 1 The Second Department of Internal Medicine, Osaka Medical and Pharmaceutical University, Takatsuki, Osaka, 569-8686, Japan.; 2 Cancer Chemotherapy Center, Osaka Medical and Pharmaceutical University, Takatsuki, Osaka, 569-8686, Japan.; 3 The Premier Departmental Research of Medicine, Osaka Medical and Pharmaceutical University, Takatsuki, Osaka, 569-8686, Japan.

**Keywords:** Advanced gastric cancer, Systemic chemotherapy, Ectopic fat deposition, Muscle quality, Prognosis

## Abstract

**Aims:** In this study, we focused on the fat ratio within psoas muscle (FRPM) and sought to clarify the impact of FRPM on overall survival (OS) in stage IV gastric cancer (GC) patients undergoing systemic chemotherapy (*n* = 79, median age = 69 years, 59 males).

**Methods:** The median FRPM was 1.67 %. Forty patients with FRPM ≥1.67 % were defined as the FRPM-high group, and the remaining 39 patients was defined as the FRPM-low group. The median PMI in male and female patients was 4.35 cm^2^/m^2^ and 2.88 cm^2^/m^2^. Thirty male patients with PMI ≥4.35 cm^2^/m^2^ and 10 female patients with PMI ≥2.88 cm^2^/m^2^ was defined as the PMI-high group, and the remaining 39 patients was defined as the PMI-low group.

**Results:** The 1-, 2- and 3- year cumulative OS rate for all cases was 70.8%, 24.3% and 14.6%. The proportion of ECOG-PS 2 or 3 in patients with FRPM-high and FRPM-low was 17.5% (7/40) and 2.6% (1/39). The 1-, 2- and 3- year cumulative OS rate in patients with FRPM-high and FRPM-low was 67.3%, 14.3% and 7.6% in the FRPM-high group and 74.8%, 40.5% and 32.4% in the FRPM-low group (*P* = 0.0341). The 1-, 2- and 3- year cumulative OS rate in patients with PMI-high and PMI-low was 86.7%, 40.4% and 30.0% in the PMI-high group and 55.8%, 12.8% and 6.4% in the PMI-low group (*P* < 0.0001). In the multivariate analysis of factors associated with OS, PMI (*P* = 0.0047) and FRPM (*P* = 0.0019) were independent predictors for the OS.

**Conclusion:** Higher FRPM can be associated with decreased physical activity, and not only skeletal muscle mass but also skeletal muscle function can be an essential prognostic factor in stage IV GC patients undergoing systemic chemotherapy.

## Introduction

Systemic chemotherapy for unresectable advanced or recurrent gastric cancer (GC) has been highly effective in reducing tumor size due to recent therapeutic advances 1-3. However, radical cure by systemic chemotherapy for advanced GC is difficult at present. Based on the results of Japanese or international clinical trials, the median survival time (MST) for unresectable advanced or recurrent GC is roughly 15 months 1, 4. In the treatment of advanced GC, the therapeutic goals are to improve clinical symptoms associated with cancer progression, to delay the onset of clinical symptoms, to delay tumor progression, and to prolong survival. Patients with advanced malignancy are prone to muscle protein loss due to muscle proteolysis-inducible substances and inflammatory cytokines released by the tumor cells 5, 6. Advanced GC is no exception and is associated with a high rate of sarcopenia, defined by a loss of quantity and quality of skeletal muscle 7. Sarcopenia in patients with advanced GC is also correlated with poor prognosis 8.

In addition to subcutaneous and visceral fat, the third type of fat that accumulates in the body is ectopic fat 9. Ectopic fat is deposited in tissues where excessive accumulation of fat is not originally desired, such as skeletal muscle, liver, and vessel walls, and affects insulin sensitivity and lipid metabolism 9-11. In Asians, including Japanese, ectopic fat tend to accumulate at a higher rate than in Westerners, even if they are not so overweight 12. Ectopic fat infiltration into skeletal muscle is also known as myosteatosis 13, 14. Ectopic fat is considered more dangerous than visceral fat because the accumulation of ectopic fat in organs is thought to worsen organ function itself. Ectopic accumulation of fat within skeletal muscle not only contributes to muscle weakness, but also interferes with the supply of nutrients to myofibers and can be associated with disease progression 15, 16. A recent study reported that ectopic fat in skeletal muscle and liver can be linked to poorer survival in patients with colorectal liver metastases 17.

The impact of ectopic fat deposition in skeletal muscle (i.e., changes in muscle quality) on clinical outcomes in GC patients has been reported in several studies 18-24. As far as we are aware, however, the data regarding muscle quality on survival in patients with stage IV GC undergoing systemic chemotherapy are currently scarce. Intramuscular adipose tissue is associated with CT density of muscle 19-24. Previous studies have mainly focused on CT density of muscle 19-24. In this study, we focused on the fat ratio within psoas muscle (FRPM) considering CT density for fat within psoas muscle, and sought to clarify these clinical research questions.

## Patients and methods

### Patients and our study

Between February 2017 and March 2021, a total of 79 patients with histologically confirmed stage IV GC receiving at least one systemic chemotherapy in our hospital can be found in our medical record, which were subject to our current analysis. Our primary endpoint was overall survival (OS). In our hospital, treatment strategy and chemotherapeutic regimen were carefully selected through discussion with surgeons and oncologists based on the current guidelines for GC. Factors relevant to the OS were retrospectively analyzed by univariate and multivariate analysis. Ascites was graded (none, mild, moderate and severe) by the CT images. The ethics committee of Osaka Medical and Pharmaceutical University hospital provided ethical approval (approval number, 2021-014).

### Psoas muscle index (PMI) and our proposed FRPM

**Figure 1:**
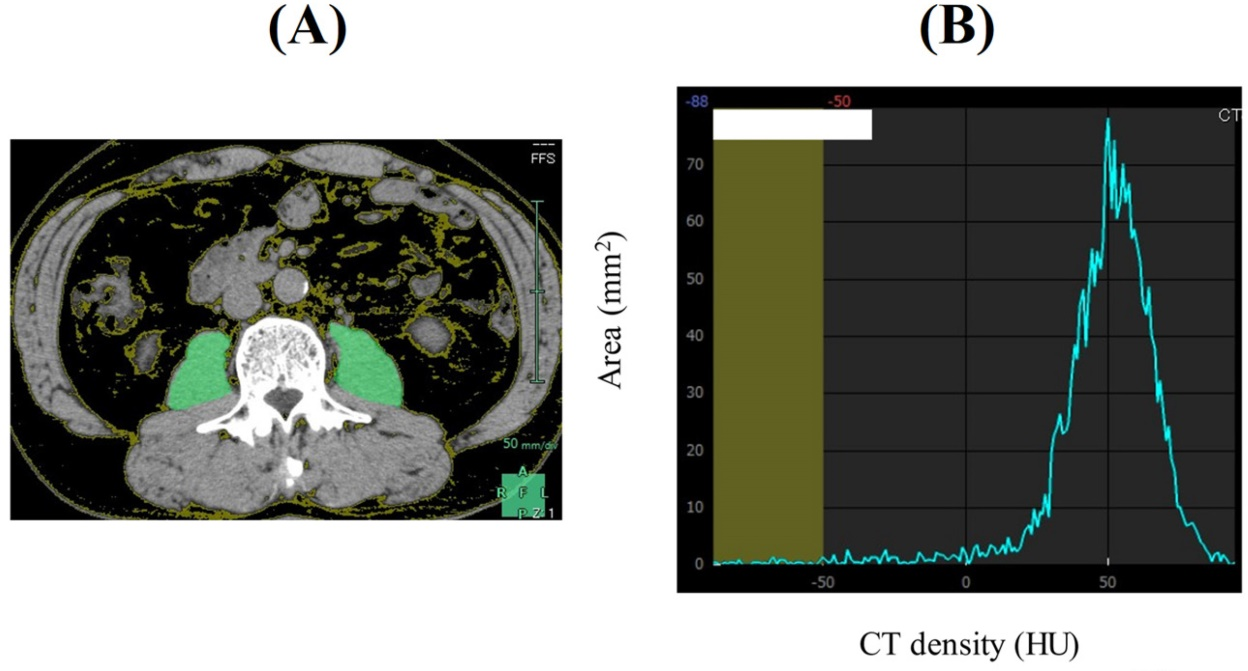
A representative CT image at the lumbar three level (Figure 1A). The sum of the areas within the left and right psoas muscles corresponding to fat (cm^2^, CT density: -200 HU to -50 HU) was calculated by Vincent®. In this case, area under the curve corresponding to CT density less than -50 HU is 13.71 mm^2^. The whole area under the curve is 2207.15 mm^2^. Thus, FRPM is calculated as 0.67% (Figure 1B).

PMI was defined as sum of bilateral psoas muscle mass calculated by Vincent® (SYNAPSE VINCENT, Fuji Film medical corporation, Tokyo, Japan) at the lumbar three (L3) level on the CT images (at the time of initial chemotherapy) divided by height squared (cm^2^/m^2^). Our proposed FRPM at the L3 level (at the time of initial chemotherapy) was calculated as follows: fat mass within bilateral psoas muscle at the L3 level calculated by Vincent® (the sum of the areas within the left and right psoas muscles corresponding to fat (cm^2^)) divided by bilateral psoas muscle mass calculated by Vincent® (cm^2^) × 100 (%) (Figure 1). CT density for fat is defined as -200 Hounsfield Units (HU) to -50 HU. To minimize measurement bias, a single trained researcher (T. I.) identified and measured psoas muscle mass and fat within psoas muscle.

### Statistics

For analyzing continuous variables, the appropriate statistical method among Student's t test, Mann-Whitney U test and Spearman's rank correlation coefficient (*r_s_*) was chosen in order to compare 2 groups. For analyzing categorical variables, Pearson χ^2^ test was applied in order to evaluate between-group difference. For analyzing the significance of prognostic parameters, continuous parameters were divided into 2 groups at the median, and transformed into nominal variables. For the estimation of cumulative OS rate, we used the Kaplan-Meier method and tested by the log-rank test. A Cox proportional hazard model was applied for the multivariate analysis of parameters with a *P* value <0.05 in the univariate analysis. The observation period was defined as the time interval between the date of initial chemotherapy and the date of death or the last date of confirmed survival. In the data presentation, *n* (%) or median (interquartile range (IQR)) was used. Statistical software was JMP ver. 15 (SAS Institute Inc., Cary, NC), with a *P* value = 0.05 as the significance level.

## Results

### Patient baseline features

Baseline features for all study subjects (*n* = 79, 59 men and 20 women, median (IQR) age = 69 (59-74) years) are summarized in Table 1. All patients were histologically confirmed GC patients. The median (IQR) body mass index (BMI) was 21.5 (19.7-23.2) kg/m^2^. Eastern Cooperative Oncology Group Performance Status (ECOG-PS) 0 was found in 38 cases (48.1%), 1 in 33 (41.8%), 2 in 7 (8.9%), and 3 in 1 (1.3%). In terms of Human epidermal growth factor receptor 2 (HER2) status, 10 patients (12.7%) had HER2-positive GC, 59 (74.7%) had HER2-negative GC and the remaining 10 patients (12.7%) had unknown HER2 status. In terms of ascites, 45 patients (57.0%) had no ascites, 19 (24.1%) had mild ascites, 9 (11.4%) had moderate ascites and 6 (7.6%) had severe ascites. The median (IQR) FRPM was 1.67 (0.64-3.14) %. Forty patients with FRPM ≥1.67% were defined as the FRPM-high group, and the remaining 39 patients was defined as the FRPM-low group. The median (IQR) PMI in male and female patients was 4.35 (3.83-5.85) cm^2^/m^2^ and 2.88 (2.26-4.34) cm^2^/m^2^. Thirty male patients with PMI ≥4.35 cm^2^/m^2^ and 10 female patients with PMI ≥2.88 cm^2^/m^2^ was defined as the PMI-high group, and the remaining 39 patients was defined as the PMI-low group.

### Initial systemic chemotherapeutic regimen

In terms of initial chemotherapeutic regimen, S-1 monotherapy was performed in 3 cases, paclitaxel monotherapy in 1, S-1 plus trastuzumab therapy in 1, oxaliplatin plus capecitabine (XELOX) in 2, capecitabine plus cisplatin (XP) in 1, S-1 plus oxaliplatin (SOX) therapy in 27, SOX plus trastuzumab therapy in 6, SOX plus nivolumab therapy in 2, oxaliplatin plus leucovorin plus 5-fluorouracil therapy (FOLFOX) in 26, XP plus pembrolizumab therapy in 1, docetaxel plus oxaliplatin plus S-1 (DOS) in 3, and S-1 plus irinotecan plus oxaliplatin (TIROX) in 2. The remaining patients participated in double-blinded randomized clinical trials regarding immune checkpoint inhibitors such as nivolumab and pembrolizumab.

### The cumulative OS rate according to the FRPM

**Figure 2:**
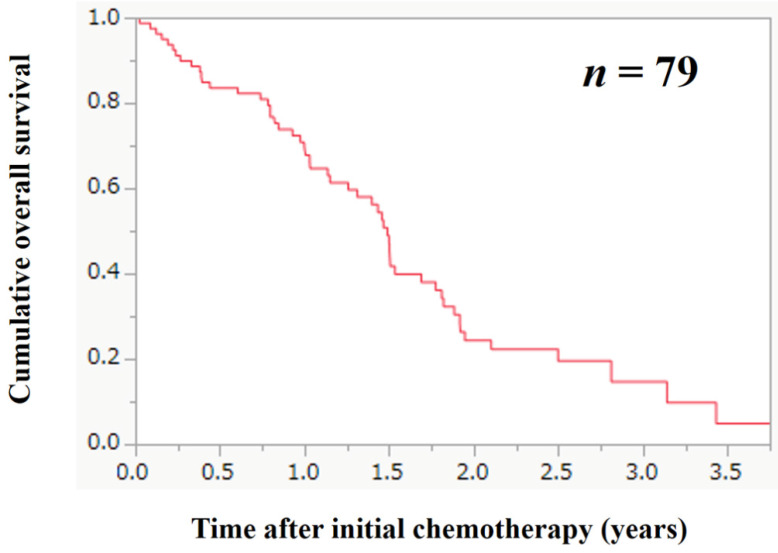
Cumulative overall survival (OS) for all cases (*n* = 79). The 1-, 2- and 3- year cumulative OS rate for all cases was 70.8%, 24.3% and 14.6%. The median survival time for all cases was 1.47 years.

**Figure 3:**
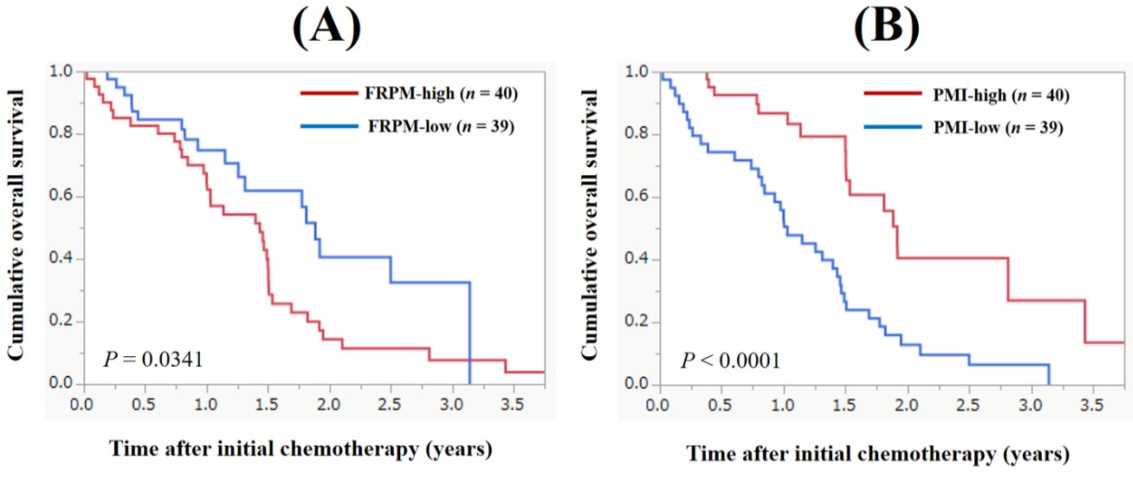
Cumulative overall survival according to the fat ratio within psoas muscle (FRPM, Figure 3A) and psoas muscle index (PMI, Figure 3B).

The median follow-up period in this study was 1.11 years. During the follow-up period, 53 patients (67.1%) died. All deaths were GC-related deaths. The 1-, 2- and 3- year cumulative OS rate for all cases was 70.8%, 24.3% and 14.6%. The MST for all cases was 1.47 years (Figure 2). The 1-, 2- and 3- year cumulative OS rate in patients with FRPM-high and FRPM-low was 67.3%, 14.3% and 7.6% in the FRPM-high group and 74.8%, 40.5% and 32.4% in the FRPM-low group (*P* = 0.0341, Figure 3A).

### The cumulative OS rate according to the PMI

The 1-, 2- and 3- year cumulative OS rate in patients with PMI-high and PMI-low was 86.7%, 40.4% and 30.0% in the PMI-high group and 55.8%, 12.8% and 6.4% in the PMI-low group (*P* < 0.0001, Figure 3B).

### Survival according to the FRPM and the PMI in male and female

**Figure 4:**
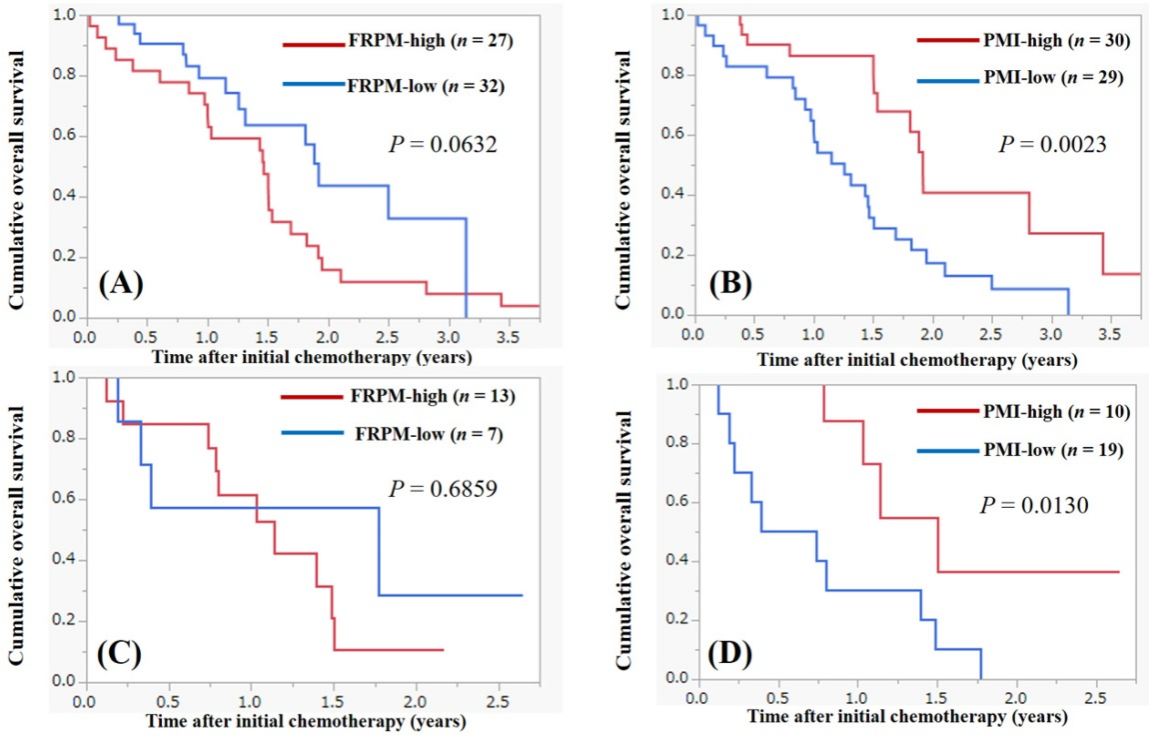
Survival according to the FRPM and the PMI in male and female. (A) Survival according to the FRPM in male. (B) Survival according to the PMI in male. (C) Survival according to the FRPM in female. (D) Survival according to the PMI in female.

Survival analysis according to the FRPM and the PMI in male is shown in Figure 4A and 4B. In male, patients with FRPM-high had tendency for poorer survival compared with those with FRPM-low (*P* = 0.0632, Figure 4A). Patients with PMI-high had significantly better survival compared with those with PMI-low (*P* = 0.0023, Figure 4B). In female, the difference of survival between FRPM-high and low did not reach significance (*P* = 0.6859, Figure 4C), while patients with PMI-high had significantly better survival compared with those with PMI-low (*P* = 0.0130, Figure 4D).

### Uni- and multivariate analysis of variables for the OS

In the univariate analysis of variables for the OS for all cases, ECOG-PS 0 (*P =* 0.0059), presence of ascites (*P* = 0.0106), C reactive protein (CRP) ≥0.5 mg/dl (*P* = 0.0121), PMI-high (*P* < 0.0001), and FRPM-high (*P* = 0.0341) were significant factors (Table 2). In the multivariate Cox regression analysis for the OS, PMI-high (*P* = 0.0047), and FRPM-high (*P* = 0.0019) were independent predictors for the OS.

### Comparison of baseline data in patients with FRPM-high and FRPM-low

In comparison of baseline data in patients with FRPM-high and FRPM-low, the distribution of ECOG-PS was significantly different between the two groups (*P* = 0.0297). The proportion of ECOG-PS 0 in patients with FRPM-high and FRPM-low was 40% (16/40) and 56.4% (22/39). The proportion of ECOG-PS 2 or 3 in patients with FRPM-high and FRPM-low was 17.5% (7/40) and 2.6% (1/39). Age in the FRPM-high group tended to be significantly higher than that in the FRPM-low group (*P* = 0.0794). Estimated glomerular filtration rate (eGFR) in the FRPM-high group was significantly lower than that in the FRPM-low group (*P* = 0.0365) (Table 3).

### Comparison of baseline data in patients with PMI-high and PMI-low

In comparison of baseline data in patients with PMI-high and PMI-low, significant difference was not found in any clinical parameter, but BMI in the PMI-high group tended to be significantly higher than that in the PMI-low group (*P* = 0.0812) (Table 4).

### Correlation between PMI and FRPM in male and female patients

**Figure 5:**
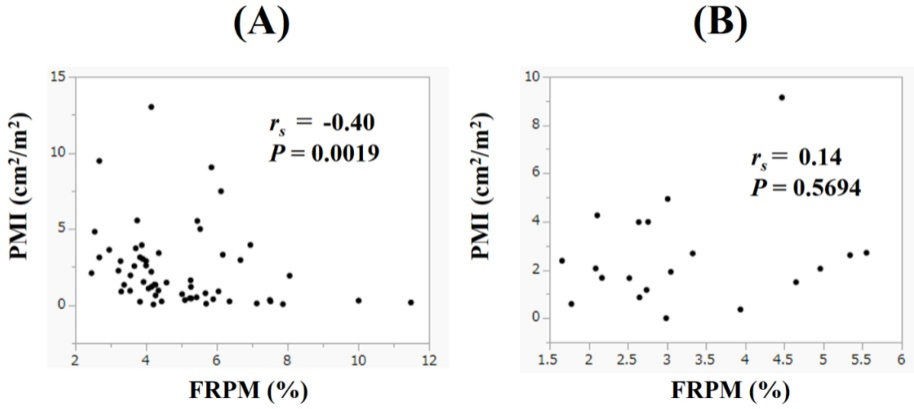
Correlation between PMI and FRPM in male (Figure 5A) and female (Figure 5B) patients.

In male, PMI had the significant negative correlation with FRPM (*r_s_* = -0.40, *P* = 0.0019), while in female, such tendency was not found (*r_s_* = 0.14, *P* = 0.5694) (Figure 5A and 5B).

## Discussion

Research on skeletal muscle has developed rapidly in recent years. Ectopic fat accumulation in skeletal muscle interferes with muscle function 9. Thus, the degree of ectopic fat accumulation may reflect skeletal muscle function and severity of sarcopenia 9. One of the mechanisms of insulin resistance in skeletal muscle is impaired insulin signaling, which is thought to be caused by ectopic fat accumulation 25, 26. In this study, we focused on the FRPM and investigated its clinical significance in patients with stage IV GC undergoing systemic chemotherapy. As far as we know, there are few data regarding the impact of the severity of fat deposition within psoas muscle on OS in stage IV GC patients receiving systemic chemotherapy. Previous studies have mainly focused on CT density of muscle itself (i.e., intramuscular adipose tissue content) 19-24. In this regard, we would like to emphasize the novelty of our proposed FRPM. As stated earlier, myosteatosis is ectopic adipose tissue infiltration into skeletal muscle 13, 14, while we primarily focused on adipose tissue itself within psoas muscle. The MST in our data was 1.47 years, which is in line with the previous data 1, 4.

In our multivariate analysis, PMI and FRPM were independent predictors for OS. These results imply that not only muscle mass but also muscle quality is an essential predictive marker in patients with advanced GC undergoing systemic chemotherapy. Exercise intervention for improving muscle function can be recommended for patients with decreased PMI and/or higher FRPM 27-29. In our data, the proportion of ECOG-PS 0 in patients with FRPM-high and FRPM-low was 40% (16/40) and 56.4% (22/39), and the proportion of ECOG-PS 2 or 3 in patients with FRPM-high and FRPM-low was 17.5% (7/40) and 2.6% (1/39), which are largely different between the two groups. Our proposed FRPM can reflect physical activity. Patients with FRPM-high tended to be significantly older than those with FRPM-low. Aging-related increase in ectopic fat deposition can be closely associated with worse health conditions 30. Patients with FRPM-high had significantly lower eGFR level than those with FRPM-low. Ectopic fat accumulation in the skeletal muscle may be linked to ectopic fat accumulation in the kidney, causing lower eGFR 31. Older age in patients with FRPM-high can cause lower eGFR.

The average minus 2 standard deviation value of PMI calculated by CT images in healthy Japanese subjects is reported to be 6.36 cm^2^/m^2^ for male and 3.92 cm^2^/m^2^ for female 32. In the present study, the median PMI in male and female was 4.35 cm^2^/m^2^ and 2.88 cm^2^/m^2^, which was significantly lower than those reported in healthy Japanese subjects. Presence of advanced GC may be associated with the current results. On the other hand, in male, PMI had the significant negative correlation with FRPM, whereas in female such tendency was not found. Gender differences in ectopic fat accumulation in skeletal muscle may be an important finding. Inflammatory markers such as CRP and neutrophil to lymphocyte ratio are reported to be prognostic markers in patients with GC 33, but in our analysis such markers were not independent factors linked to OS. This may be partly due to the fact that the subjects in this study were limited to stage IV GC patients.

In our previous study, we have reported the prognostic significance of PMI in patients with hepatocellular carcinoma undergoing radiofrequency thermal ablation and pancreatic cancer undergoing systemic chemotherapy 34, 35. PMI well predicts prognosis in patients with colorectal cancer 36. PMI can be helpful for predicting postoperative outcomes in patients with esophageal cancer undergoing surgery 37. A decreased PMI is associated with poorer prognosis in patients with metastatic hormone-naïve prostate cancer 38. A decreased PMI predicts cancer recurrence in patients with upper urinary tract urothelial carcinoma 39. The prognostic impact of PMI was also shown in this study. Indeed, measurement of PMI is clinically useful in various malignancies. Psoas muscle mass at the L3 level correlates well with total body skeletal muscle mass, and can be easily measured in a short time 40. On the other hand, psoas muscle is not symmetrical in shape, and it can take various forms 41. Clinicians should be fully aware of these.

Several limitations must be pointed out in the present analysis. First, the current study was an observational study at a single hospital with a retrospective nature. Second, the number of patients in our cohort was relatively small, but the number of deaths was 53, which can be tolerable for the multivariate analysis of OS. Third, chemotherapeutic regimens at the initial systemic chemotherapy and chemotherapeutic dose intensity were highly heterogeneous, also creating bias. Fourth, changes in body composition during systemic chemotherapy was not analyzed in this study. Fifth, we only measured the visible fat using CT scan and dedicated software, and did not take into consideration the density of the psoas muscle itself. Sixth, the readings of CT findings were done by a single radiologist. Sixth, histological factors were not entered into analysis. Finally, data for grip strength, which well reflects muscle function, were not available in this study. Thus, care should be taken in interpreting the results. However, our study results suggested that FRPM can be a useful marker as well as muscle mass for predicting prognosis in advanced GC patients receiving systemic chemotherapy.

In conclusion, we would like to emphasize that not only skeletal muscle mass but also skeletal muscle quality can be an essential prognostic factor in patients with stage IV GC undergoing systemic chemotherapy.

## Figures and Tables

**Table 1:** Baseline characteristics (*n* = 79)

	*n* or median (IQR)
Age (years)	69 (59-74)
Sex, male/female	59/20
ECOG-PS, 0/1/2/3/4	38/33/7/1/0
HER2 status, positive/negative/unknown	10/59/10
Body mass index (kg/m^2^)	21.5 (19.7-23.2)
Ascites, none/mild/moderate/severe	45/19/9/6
Alanine aminotransferase (IU/l)	18 (12-28)
C reactive protein (mg/dl)	0.5 (0.11-3.32)
eGFR (ml/min/1.73m^2^)	69 (56-84)
Serum albumin (g/dl)	3.6 (3.1-3.9)
Neutrophil count (/μl)	4810 (3407-6464)
Total lymphocyte count (/μl)	1327 (963-1739)
Neutrophil to lymphocyte ratio	3.73 (2.27-5.72)
Fat ratio within psoas muscle at the L3 level (%)	1.67 (0.64-3.14)
PMI at the L3 level (male, cm^2^/m^2^)	4.35 (3.83-5.85)
PMI at the L3 level (female, cm^2^/m^2^)	2.88 (2.26-4.34)

IQR; interquartile range, ECOG-PS; Eastern Cooperative Oncology Group Performance Status, HER2; Human epidermal growth factor receptor 2, eGFR; estimated glomerular filtration rate; PMI; psoas muscle index.

**Table 2:** Univariate and multivariate analysis of factors associated with survival for all cases

Variables	Univariate		Multivariate		
	n	*P* value	HR	95% CI	*P* value
Age ≥69 years, yes/no	40/39	0.7706			
Sex, male/female	59/20	0.0811			
ECOG-PS 0, yes/no	38/41	0.0059	1.531	0.617-3.799	0.3578
Presence of ascites, yes/no	34/45	0.0106	0.864	0.342-2.187	0.7582
BMI ≥21.5 kg/m^2^, yes/no	39/40	0.1207			
Serum albumin ≥3.6 g/dl, yes/no	43/36	0.0920			
CRP ≥0.5 mg/dl, yes/no	40/39	0.0121	0.819	0.326-2.060	0.6719
NLR ≥3.73, yes/no	40/39	0.0817			
eGFR ≥69 ml/min/1.73m^2^, yes/no	40/39	0.8701			
PMI-High, yes/no	40/39	<0.0001	6.399	1.767-23.175	0.0047
FRPM-High, yes/no	40/39	0.0341	0.170	0.056-0.521	0.0019

HR; hazard ratio, CI; confidence interval, ECOG-PS; Eastern Cooperative Oncology Group Performance Status, BMI; body mass index, CRP; C reactive protein, NLR; neutrophil to lymphocyte ratio, eGFR; estimated glomerular filtration rate, PMI; psoas muscle index, FRPM; fat ratio within psoas muscle.

**Table 3:** Comparison of baseline data in patients with FRPM-high and FRPM-low

Variables	FRPM-high (*n* = 40)	FRPM-low (*n* = 39)	*P* value
Age (years)	71 (64-76)	66 (58-71)	0.0794
Sex, male/female	27/13	32 / 7	0.1370
BMI (kg/m^2^)	22.4 (20.1-23.7)	20.8 (19.6-22.1)	0.2132
EOCG-PS, 0/1/2/3	16/17/7/0	22/16/0/1	0.0297
Ascites, none/mild/moderate/severe	24/7/4/5	21/12/5/1	0.2326
Serum albumin	3.6 (3.1-3.9)	3.6 (3.1-3.9)	0.9871
CRP (mg/dl)	0.58 (0.2-3.1)	0.37 (0.07-3.32)	0.5813
NLR	4.14 (2.22-6.53)	3.49 (2.44-4.87)	0.3244
ALT (IU/l)	17 (10-24)	19 (13-37)	0.1066
eGFR (ml/min/1.73m^2^)	65 (54-76)	78 (56-89)	0.0365

Data are presented as number or median (interquartile range). FRPM; fat ration within psoas muscle, BMI; body mass index, ECOS-PS; Eastern Cooperative Oncology Group Performance Status, CRP; C reactive protein, NLR; neutrophil to lymphocyte ratio, ALT; alanine aminotransferase, eGFR; estimated glomerular filtration rate.

**Table 4:** Comparison of baseline data in patients with PMI-high and PMI-low

Variables	PMI-high (*n* = 40)	PMI-low (*n* = 39)	*P* value
Age (years)	67 (58-73)	70 (62-75)	0.2158
Sex, male/female	30/10	29/10	1.000
BMI (kg/m^2^)	21.6 (20.3-22.9)	21.0 (18.1-23.4)	0.0812
EOCG-PS, 0/1/2/3	22/16/2/0	16/17/5/1	0.3545
Ascites, none/mild/moderate/severe	24/10/4/2	21/9/5/4	0.7969
Serum albumin (g/dl)	3.6 (3.2-3.9)	3.6 (3.1-3.9)	0.3930
CRP (mg/dl)	0.60 (0.11-3.29)	0.49 (0.11-3.32)	0.8260
NLR	3.61 (2.47-4.73)	4.0 (2.21-6.06)	0.5192
ALT (IU/l)	18 (12-27)	18 (12-31)	0.9688
eGFR (ml/min/1.73m^2^)	72 (56-84)	67 (54-85)	0.8151

Data are presented as number or median (interquartile range). PMI; psoas muscle index, BMI; body mass index, ECOG-PS; Eastern Cooperative Oncology Group Performance Status, CRP; C reactive protein, NLR; neutrophil to lymphocyte ratio, ALT; alanine aminotransferase, eGFR; estimated glomerular filtration rate.

## Abbreviations

GC: gastric cancer
MST: median survival time
FRPM: fat ratio within psoas muscle
OS: overall survival
PMI: psoas muscle index
HU: Hounsfield Unit
IQR: interquartile range
ECOG-PS: Eastern Cooperative Oncology Group Performance Status
BMI: body mass index
HER2: Human epidermal growth factor receptor 2
XP: capecitabine plus cisplatin
SOX: S-1 plus oxaliplatin
CRP: C reactive protein
eGFR: estimated glomerular filtration rate
